# Pulmonary Rehabilitation Can Improve the Functional Capacity and Quality of Life for Pneumoconiosis Patients: A Systematic Review and Meta-Analysis

**DOI:** 10.1155/2020/6174936

**Published:** 2020-07-28

**Authors:** Hulei Zhao, Yang Xie, Jiajia Wang, Xuanlin Li, Jiansheng Li

**Affiliations:** ^1^Longhua Hospital Shanghai University of Traditional Chinese Medicine, Shanghai, China; ^2^Co-Construction Collaborative Innovation Center for Chinese Medicine and Respiratory Diseases by Henan & Education Ministry of P.R. China, Zhengzhou, China; ^3^Department of Respiratory Diseases, The First Affiliated Hospital of Henan University of Chinese Medicine, Zhengzhou, China

## Abstract

This study evaluated the efficacy and safety of pulmonary rehabilitation (PR) for pneumoconiosis. We systematically searched PubMed, Embase, The Cochrane Library, Web of Science, SinoMed, CNKI, VIP databases and Wanfang Data from their inception to June 1, 2019. A systematic review and meta-analysis of randomized controlled trials (RCTs) of PR for pneumoconiosis was conducted and reported in compliance with the Preferred Reporting Items for Systematic Reviews and Meta-Analyses (PRISMA). Two reviewers independently screened literature, extracted data, and assessed bias risk. All statistical analyses were performed using the RevMan software. Sixteen RCTs with 1307 subjects were ultimately included for analysis. Compared with routine treatment, PR was able to improve the 6-minute walking distance (mean difference (MD) 69.10, 95% confidence interval (CI) 61.95–76.25); the 36-Item Short Form Health Survey total score (MD 17.60, 95% CI 13.59–21.61); physical function score (MD 15.45, 95% CI 3.20–27.69); role physical score (MD 17.87, 95% CI 12.06–23.69); body pain score (MD 14.34, 95% CI 10.33–18.36); general health score (MD 20.86, 95% CI 16.87–24.84); vitality score (MD 11.66, 95% CI 0.18–23.13); social function score (MD 9.67, 95% CI 1.27–18.08); mental health score (MD 20.60, 95% CI 13.61–27.59); forced vital capacity (FVC) (MD 0.20, 95% CI 0.12–0.29); forced expiratory volume in 1 s (FEV1) (MD 0.23, 95% CI 0.09–0.38); FEV1% (MD 5.19, 95% CI 1.48–8.90); maximal voluntary ventilation (MD 4.47, 95% CI 1.14–7.81); reduction in the St. George's Respiratory Questionnaire score (MD -9.60, 95% CI -16.40 to -2.80); and the modified Medical Research Council Scale score. Furthermore, PR did not increase the FEV1/FVC (MD 3.61, 95% CI -3.43 to 10.65), nor the emotional score (MD 6.18, 95% CI -23.01 to 35.38) compared with the control. We found no reports of adverse events associated with PR. Thus, to some extent, PR can improve functional capacity and quality of life in patients with pneumoconiosis. However, these results should be interpreted with caution because of high heterogeneity. This trial is registered with registration number CRD42018095266.

## 1. Introduction

Pneumoconiosis refers to a group of occupational lung diseases characterized by diffuse fibrosis of the lung tissue. It is caused mainly by long-term inhalation and deposition of mineral dust, with varying levels of pathogenicity, into the lungs during occupational activities [1–6]. There are 12 forms of occupational pneumoconiosis prescribed by the law in China, of which silicosis and coal worker pneumoconiosis are the most common. Patients with pneumoconiosis are typically affected by cough, expectoration, chest distress, and dyspnea [1]. Pneumoconiosis is a progressive and irreversible but preventable lung disease [7, 8]. It compromises personal health and can cause serious harm among families [9, 10].

According to the statistical bulletin on the development of health and health services in China in 2018, 19 468 cases of occupational pneumoconiosis were reported, accounting for 82.85% of a total of 23 497 cases of occupational diseases [11]. The prevalence of silicosis from 2002 to 2016 was 12.7% [12]. The number of new cases of pneumoconiosis in China showed an upward trend from 2000 to 2016 and a slight decline after 2016 [13, 14]. At present, more than 870 000 cases of pneumoconiosis have been reported in China [15], with a mortality as high as 31.2% [16]. Particularly, long-term exposure to silica dust is associated with substantially increased mortality [17]. However, various reports indicate that the actual number of patients with pneumoconiosis in China has exceeded 6 million [18].

No specific drugs or other treatment methods currently exist for pneumoconiosis [19]. Therefore, the management of the condition should first strengthen a comprehensive approach to overall health and active interventions (including symptomatic treatment, complication/combination treatment, and rehabilitative treatment) to reduce pain, delay progression of the disease, and prolong the life of the patient [1]. Comprehensive intervention has been clearly demonstrated to alleviate dyspnea and improve exercise performance and the health-related quality of life (QoL) [20]. Comprehensive interventions, including exercise training, education and behavioral change, and pulmonary rehabilitation (PR), have been widely used in the treatment of respiratory diseases and can effectively yield sustained improvement in functional capacity and reduced need for clinical care [21–26].

Exercise training can improve physical ability and the quality of life in patients with nonmalignant respiratory diseases. However, generally, fewer patients with pneumoconiosis have been included in previous relevant studies [27]. Recently, several randomized controlled trials (RCTs) of PR in the treatment of pneumoconiosis have also been reported. To further evaluate the efficacy and safety of PR for pneumoconiosis, we included a comparatively greater number of patients with pneumoconiosis and conducted a meta-analysis.

## 2. Materials and Methods

This systematic review and meta-analysis was conducted in accordance with the Cochrane Handbook for Systematic Reviews of Interventions [28], and reported in compliance with the Preferred Reporting Items for Systematic Reviews and Meta-Analyses (PRISMA) [29] (Supplementary data: Table S1). The study protocol was prospectively registered in the International Prospective Register of Systematic Reviews (PROSPERO) registry (CRD42018095266) and published [30].

### 2.1. Eligibility Criteria

#### 2.1.1. Study Type and Participants

All published RCTs with a parallel, cluster, or crossover design of PR for patients with pneumoconiosis were included. Only studies in which the authors specifically noted a diagnosis of pneumoconiosis were considered for inclusion.

#### 2.1.2. Intervention

The intervention on which we were focused was PR, based on exercise training. Furthermore, the intervention may or may not have included health education, nutritional intervention, and/or psychosocial support.

#### 2.1.3. Outcomes

*(1) Primary Outcomes*. The primary outcome measures were functional capacity and health-related QoL, as measured by the 6-minute walking distance (6MWD) and St. George's Respiratory Questionnaire (SGRQ).

*(2) Secondary Outcomes*. The secondary outcome measures were pulmonary function, modified Medical Research Council (mMRC) dyspnea scale, 36-Item Short Form Health Survey (SF-36), acute exacerbations, and adverse events.

### 2.2. Search Strategy

We conducted a systematic search of PubMed, Embase, The Cochrane Library, Web of Science, SinoMed, CNKI, VIP databases and Wanfang Data from their inception to June 1, 2019, using search terms comprising medical subject headings (MeSH) and free-text terms. We developed detailed search strategies for each electronic database, without language restrictions, to attempt to identify all eligible studies. The search strategy for PubMed is shown in Table 1. The search terms were modified and adapted in other databases. We also conducted a search on the websites of ClinicalTrials.gov, the WHO International Clinical Trials Registry platform, Chinese Clinical Trial Registry, and Conference Proceedings Index (Web of Science Core Collection) to identify additional ongoing or unpublished studies. References of the retrieved articles and previous reviews were also checked manually to identify additional potentially eligible studies.

### 2.3. Trial Selection

**Figure 1:**
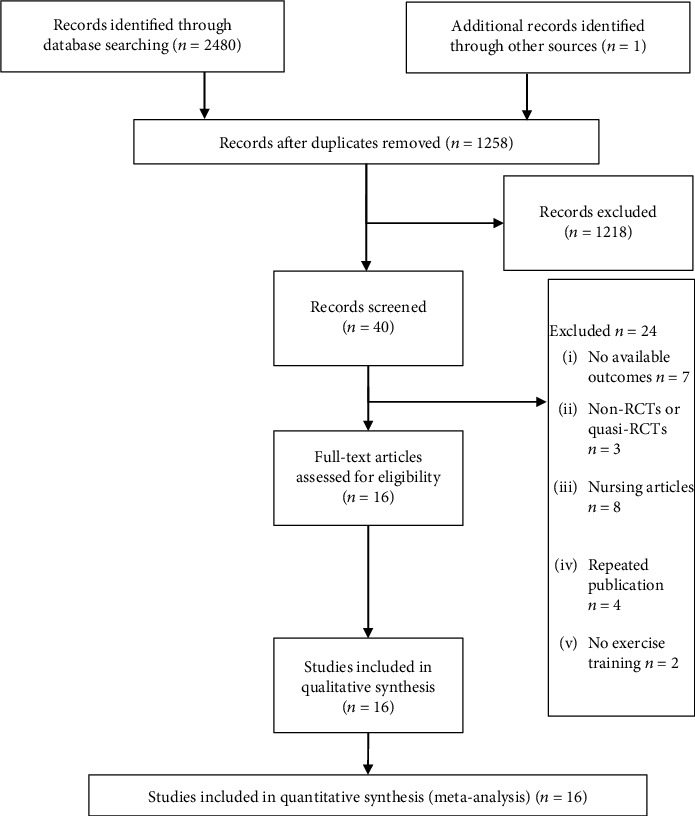
Selection of RCTs for the meta-analysis.

After records were imported into the EndNote reference management software (Clarivate Analytics), duplicate records were removed. Two reviewers (J-JW and X-LL) independently examined titles and abstracts and selected all potentially eligible studies. Full-text articles were then obtained and reviewed independently, based on the inclusion criteria. We resolved all disagreements by consensus, and a third reviewer (H-LZ) acted as an arbiter when consensus could not have been achieved. Details of the selection process are presented in Figure 1.

### 2.4. Data Extraction

Two investigators (J-JW and X-LL) independently extracted data from the included studies. Disagreements were solved by consultation with the third reviewer (H-LZ). Structured and standardized data extraction sheets were completed for every study included in the review. Those sheets included details of the authors, year of publication, study design, characteristics of participants, intervention, comparator, and outcomes.

### 2.5. Risk-of-Bias Assessment

Methodological quality was independently assessed using the Cochrane Collaboration's tool for assessing the risk of bias [31–33]. The assessment details included sequence generation, allocation concealment, blinding of participants and personnel, blinding of outcome assessors, incomplete outcome data, selective reporting, and other sources of bias. Each domain was assessed as “low risk,” “high risk,” or “unclear risk,” according to the description details of the eligible studies. We summarized the risk of bias and solved differences in author interpretation of the data through discussion.

### 2.6. Quality of Evidence Assessment

We also evaluated the quality of evidence for the outcome measures using the Grading of Recommendations Assessment, Development and Evaluation (GRADE) system [34]. The GRADE system divides the quality of evidence into four levels: high, medium, low, and very low. A summary table was prepared using the GRADE profiler (GRADEpro, version 3.6).

### 2.7. Data Analysis

For continuous data, we calculated the mean differences (MD) with 95% confidence intervals (CI). When different units of measurement were employed, each unit was converted to the most common. For example, we converted the “month” or “year” into “weeks”.

The *χ*^2^ test for heterogeneity was performed. We used the random effects model across all analyses, to address clinical heterogeneity of the included population, as well as variations in treatment duration. Publication bias was assessed via a funnel plot.

We also performed subgroup analyses according to different intervention forms (exercise training and health education or exercise training and health education combined with other measures) and durations of PR (<3 months or ≥3 months). We excluded studies in succession and compared the results through sensitivity analysis. All statistical analyses were performed using the Review Manager (RevMan) software [35]. Publication bias was assessed by Begg's test and Egger's test with the STATA software version 12.0 [36].

## 3. Results

### 3.1. Results of the Search

A total of 2481 studies were retrieved following both electronic and manual searches. After the removal of the duplicates and screening of the titles and abstracts, 40 articles were deemed potentially eligible. After reviewing the full-text articles, 16 trials [37–52] were included in the final analysis. The screening process is summarized in a flow diagram (Figure 1).

### 3.2. Characteristics of the Included Studies

This review included 16 RCTs published from 2009 to 2019, with a minimum sample size of 10 and a maximum of 200. The total numbers of patients in the PR group and control group were 655 and 652, respectively. The duration of treatment ranged from 1.5 to 12 months. The characteristics of the included studies are presented in Table 2.

### 3.3. Assessment of Risk of Bias

**Figure 2:**
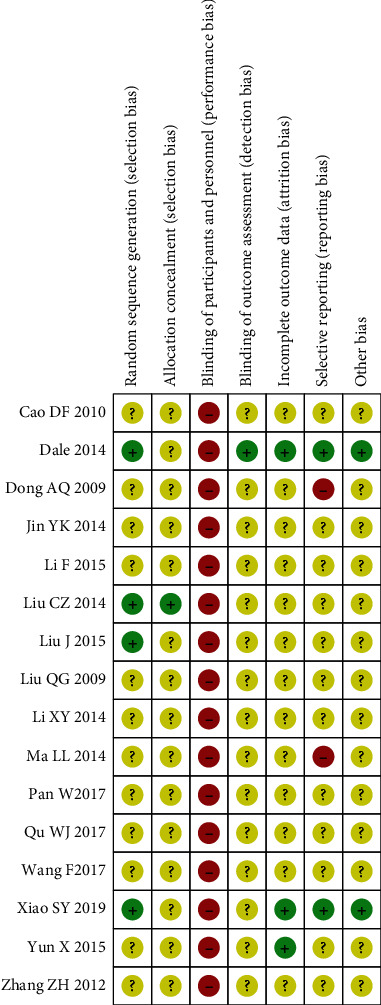
Risk-of-bias graph. +: low risk; −: high risk; ?: uncertain risk.

**Figure 3:**
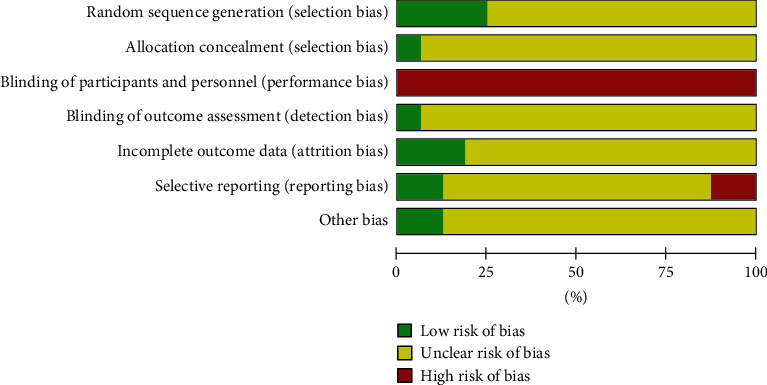
Risk-of-bias summary.

Of the 16 included studies, 14 were judged to show unclear risk and 2, high risk. Four studies described randomization methods, of which two used a random table of numbers, one used a lottery, and one used a computer-generated randomization program. One study used allocation concealment via a lottery. Participants were not blinded to the specific intervention in any of the studies. In one study, the assessor was blinded. Three studies reported loss to follow-up and dropouts. Two studies did not report the primary outcomes. The results of risk of bias assessment are summarized in Figures 2 and 3. The statements for risk of bias for all 16 included studies are shown in Table 3.

### 3.4. Primary Outcomes

#### 3.4.1. 6MWD

**Figure 4:**
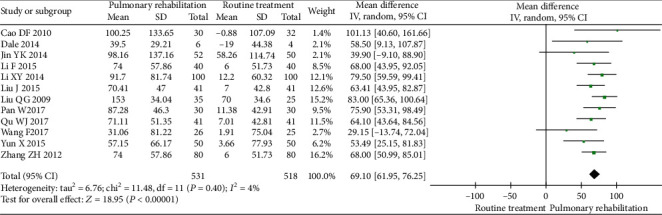
Forest plot showing the effect of PR on the 6MWD for pneumoconiosis.

Twelve studies were included in the meta-analysis. The between-study heterogeneity was low for the 6MWD. The results showed that PR could significantly increase the 6MWD (MD 69.10, 95% CI 61.95 to 76.25, *P* < 0.001, *I*^2^ = 4%) compared with the control (Figure 4).

#### 3.4.2. SGRQ

**Figure 5:**
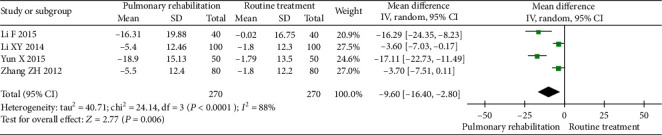
Forest plot showing the effect of PR on the SGRQ score for pneumoconiosis.

Four studies were included in the meta-analysis. The between-study heterogeneity was high for the SGRQ. The results showed that PR could reduce the SGRQ score (MD -9.60, 95% CI -16.40 to -2.80, *P* = 0.006, *I*^2^ = 88%) compared with the control (Figure 5).

### 3.5. Secondary Outcomes

#### 3.5.1. Forced Vital Capacity (FVC)

**Figure 6:**
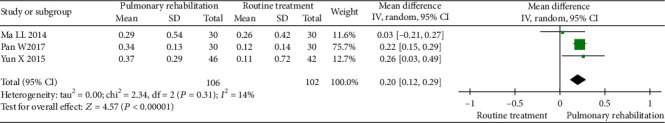
Forest plot showing the effect of PR on the FVC for pneumoconiosis.

Three studies were included in the meta-analysis. The between-study heterogeneity was low for FVC. The results showed that PR could increase the FVC (MD 0.20, 95% CI 0.12 to 0.29, *P* < 0.001, *I*^2^ = 14%) compared with the control (Figure 6).

#### 3.5.2. Forced Expiratory Volume in 1 s (FEV1)

**Figure 7:**
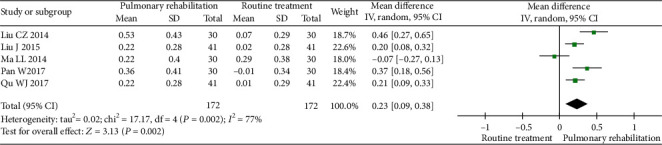
Forest plot showing the effect of PR on the FEV1 for pneumoconiosis.

Five studies were included in the meta-analysis. The between-study heterogeneity was high for the FEV1. The results showed that PR could significantly increase the FEV1 (MD 0.23, 95% CI 0.09 to 0.38, *P* = 0.002, *I*^2^ = 77%) compared with the control (Figure 7).

#### 3.5.3. FEV1%

**Figure 8:**
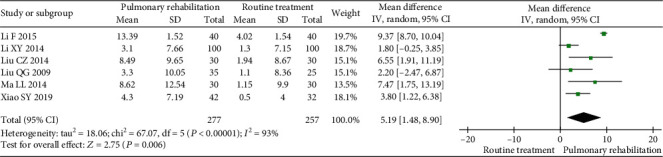
Forest plot showing the effect of PR on the FEV1% for pneumoconiosis.

Six studies were included in the meta-analysis. The between-study heterogeneity was high for the FEV1%. The results showed that PR could significantly increase the FEV1% (MD 5.19, 95% CI 1.48 to 8.90, *P* = 0.006, *I*^2^ = 93%) compared with the control (Figure 8).

#### 3.5.4. FEV1/FVC

**Figure 9:**
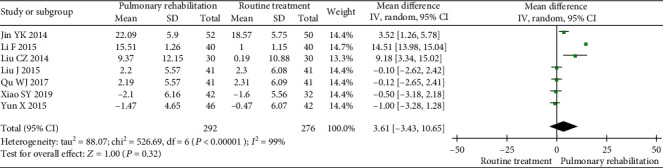
Forest plot showing the effect of PR on the FEV1/FVC for pneumoconiosis.

Seven studies were included in the meta-analysis. The between-study heterogeneity was high for the FEV1/FVC. The results showed that PR was not able to increase the FEV1/FVC (MD 3.61, 95% CI -3.43 to 10.65, *P* = 0.32, *I*^2^ = 99%) compared with the control (Figure 9).

#### 3.5.5. Maximal Voluntary Ventilation (MVV)

**Figure 10:**
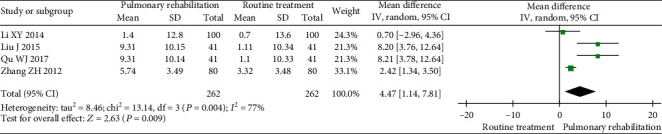
Forest plot showing the effect of PR on the MVV for pneumoconiosis.

Four studies were included in the meta-analysis. The between-study heterogeneity was high for the MVV. The results showed that PR could significantly increase the MVV (MD 4.47, 95% CI 1.14 to 7.81, *P* = 0.009, *I*^2^ = 77%) compared with the control (Figure 10).

#### 3.5.6. mMRC

**Figure 11:**
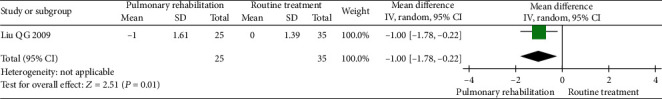
Forest plot showing the effect of PR on the mMRC for pneumoconiosis.

One study was included in the meta-analysis. The results showed that PR could reduce the mMRC compared with the control (Figure 11).

#### 3.5.7. SF-36

**Figure 12:**
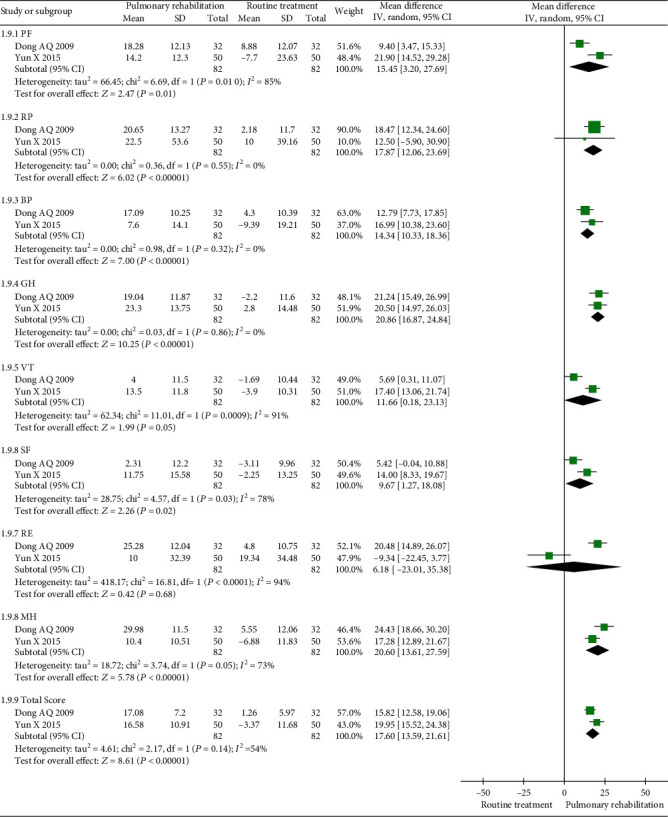
Forest plot showing the effect of PR on the SF-36 for pneumoconiosis.

Two studies were included in the meta-analysis. The between-study heterogeneity was high for the SF-36 total score and the physical function (PF), vitality (VT), social function (SF), mental health (MH), and emotional (RE) scores. Furthermore, the between-study heterogeneity was low for the role physical (RP), body pain (BP), and general health (GH) scores. The results showed that PR could increase the SF-36 total score (MD 17.60, 95% CI 13.59 to 21.61, *P* < 0.001, *I*^2^ = 54%); PF score (MD 15.45, 95% CI 3.20 to 27.69, *P* = 0.01, *I*^2^ = 85%); RP score (MD 17.87, 95% CI 12.06 to 23.69, *P* < 0.001, I^2^ = 0%); BP score (MD 14.34, 95% CI 10.33 to 18.36, *P* < 0.001, *I*^2^ = 0%); GH score (MD 20.86, 95% CI 16.87 to 24.84, *P* < 0.001, *I*^2^ = 0%); VT score (MD 11.66, 95% CI 0.18 to 23.13, *P* = 0.05, *I*^2^ = 91%); SF score (MD 9.67, 95% CI 1.27 to 18.08, *P* = 0.02, *I*^2^ = 78%); and MH score (MD 20.60, 95% CI 13.61 to 27.59, *P* < 0.001, *I*^2^ = 73%) compared with the control. However, PR was not able to increase the RE score (MD 6.18, 95% CI -23.01 to 35.38, *P* = 0.68, *I*^2^ = 94%) compared with the control (Figure 12).

### 3.6. Subgroup Analysis

We conducted subgroup analysis of the intervention and duration of PR. No statistical differences were noted in the improvement of the 6MWD among different subgroups. However, statistical differences were noted in the improvement of FEV1% between exercise training plus health education and exercise training plus health education combined with other measures. Compared with exercise training plus health education, exercise training plus health education combined with other measures showed more favorable results in improving FEV1% (Table 4). Statistical differences were noted in the improvement of SGRQ and MVV between the PR duration of <3 months and the duration of ≥3 months. Compared with the PR duration of <3 months, the duration of ≥3 months showed more favorable results in improving SGRQ scores and MVV (Table 4). Results of all subgroup analyses in the meta-analysis are presented in the Supplementary materials: Figure S1-S13.

### 3.7. Sensitivity Analysis

To further confirm the robustness of the results, we conducted a sensitivity analysis. We excluded studies one at a time and compared the results of the analysis. We found no considerable change in the direction of the overall effects of the SGRQ score, FEV1, FEV1%, FEV1/FVC, or MVV. However, we noted a change in the direction of the overall effects of FVC when two studies were excluded (Pan et al., 2017; Yun et al., 2015) (Table 5).

### 3.8. Quality of Evidence

The overall quality of evidence, according to the primary outcome measures, was moderate or low. The GRADE evidence profiles of the primary outcomes are shown in Table 6.

### 3.9. Publication Bias

**Figure 13:**
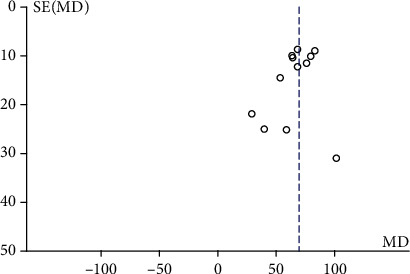
Funnel plot of the effect of PR on 6MWD.

A funnel plot was used to evaluate publication bias. We found no potential publication bias among the included trials (Figure 13) (Begg's test [*P* = 0.15] and Egger's test [*P* = 0.33]).

### 3.10. Limitations

We had hoped to also evaluate the effects of PR on acute exacerbations of pneumoconiosis and the occurrence of adverse events in patients with PR. However, the 16 studies included did not report acute exacerbations or adverse events. Therefore, the present analysis could not report them. This is a major limitation of this systematic review.

## 4. Discussion

Sixteen RCTs with 1307 subjects were ultimately included in the analysis. The main findings of the present study indicated that PR can be effective in patients with pneumoconiosis, as evidenced by the changes in the 6MWD, SGRQ, mMRC, SF-36, and pulmonary function. No adverse events were reported in any of the included studies.

The mean improvement in the 6MWD following PR was 69.10 m, which exceeds the mean improvement of 44.34 m observed in people with COPD who have undergone PR [23] and the minimal clinically important difference (24–45 m) for idiopathic pulmonary fibrosis [53]. In the present study, we also found that PR could reduce the SGRQ score compared with the control. The mean reduction in the SGRQ score following PR was 9.60, which exceeds the minimal clinically important difference for COPD [54, 55].

We also found that PR reduced the mMRC score and increased the SF-36 score, compared with the control. Regarding pulmonary function, PR was able to improve the FVC, FEV1, FEV1%, and MVV in patients with pneumoconiosis. However, these improvements were likely too small to show clinical significance and cannot induce any considerable improvement in the FEV1/FVC. These findings suggest that patients with pneumoconiosis can benefit from PR, as it can improve exercise ability and the quality of life. The results of subgroup analysis showed that prolonging the duration of PR or combining exercise training with other forms of rehabilitation, such as nutritional intervention and psychosocial support, may improve SGRQ scores and pulmonary function.

At present, pneumoconiosis is still considered an incurable and irreversible disease, and the quality of life among patients with pneumoconiosis is low [56, 57]. As the treatment goal for pneumoconiosis is to alleviate symptoms or prevent deterioration, we propose that the current guidelines should include PR as a routine treatment for pneumoconiosis. We found that PR in patients with pneumoconiosis was generally safe and well tolerated. Thus, the present evidence, at least to some extent, supports the fact that PR can be recommended for patients with pneumoconiosis.

Specific weaknesses and shortcomings of the present study must be acknowledged, which may reduce the credibility of the results. First, almost all trials were conducted in China, which may limit generalizability. Second, some weaknesses in methodology may have had considerable effects on the results. For example, only four trials clearly reported the randomization methods used. Only three trials reported attrition. Only one clinical trial was formally registered. Furthermore, we only evaluated effects after rehabilitation and found that patients may have derived more benefits by prolonging the duration of PR. However, we were unable to determine the best course of PR or evaluate the long-term effects of PR. Similar to the problems encountered in most systematic reviews, our analyses were greatly affected by heterogeneity. This is a major issue, on which we need to focus in the next steps of our research.

There are currently no effective drugs or measures to treat fibrosis due to pneumoconiosis. Our findings have important implications worldwide, for both policymakers and clinicians. We should apply PR as a routine treatment for pneumoconiosis.

## 5. Conclusions

Our results show that PR can improve patients' quality of life and functional capacity. However, the quality of the research included in the present study was low, which limits the strength of the inferences that can be drawn. This issue needs further investigation through a well-designed RCT. There is also a need for follow-up data, to demonstrate the extent to which the effects of PR are maintained over time. Future studies should also pay attention to the specific types of patients with pneumoconiosis who can derive the most benefit from PR. We should also try to determine which form of PR is the most effective and the best course of treatment. Multicenter RCTs of PR for pneumoconiosis are needed worldwide, in order to generalize the results.

Long-term exposure to mineral dust can severely compromise clearance and defense mechanisms of the respiratory system. A chronic, progressive course of disease can lead to reduced patient immunity, which can eventually lead to a variety of complications. Complications can have considerable impact on the treatment, progression, and prognosis of pneumoconiosis and can be a direct cause of patient deaths. Timely diagnosis and treatment of various complications are crucial to improving the condition of the patients, prolonging their lives, and improving their quality of life. Therefore, in our future research, we would focus on the efficacy of PR for patients with comorbidities.

The results of this study, at least to an extent, support the use of PR to improve respiratory function and the quality of life in patients with pneumoconiosis; however, we should treat the results cautiously because of the high heterogeneity among studies in the present analysis.

## Figures and Tables

**Table 1:** Search strategy for PubMed.

Number	Number search terms
#1	Pneumoconiosis [MeSH terms]
#2	Pneumoconiosis [title/abstract]
#3	Asbestosis [MeSH terms]
#4	Asbestosis [title/abstract]
#5	Silicosiss [MeSH terms]
#6	Silicosis [title/abstract]
#7	Anthracosis [MeSH terms]
#8	Anthracosis [title/abstract]
#9	Anthracosilicosis [MeSH terms]
#10	Anthracosilicosis [title/abstract]
#11	#1 OR #2 OR #3 OR #4 OR #5 OR #6 OR #7 OR #8 OR #9 OR #10
#12	Rehabilitation [MeSH terms]
#13	Rehabilitation [title/abstract]
#14	Health education [MeSH terms]
#15	Health education [title/abstract]
#16	Psychological counseling [title/abstract]
#17	Nutritional guidance [title/abstract]
#18	Baduanjin [title/abstract]
#19	Eight-section brocade [title/abstract]
#20	Respiratory training [title/abstract]
#21	Sports training [title/abstract]
#22	Exercise therapy [MeSH terms]
#23	Exercise therapy [title/abstract]
#24	Physical fitness [MeSH terms]
#25	Physical fitness [title/abstract]
#26	Physical exertion [MeSH terms]
#27	Physical exertion [title/abstract]
#28	Kinesiotherapy [title/abstract]
#29	Muscle training [MeSH terms]
#30	Muscle training [title/abstract]
#31	Physical endurance [MeSH terms]
#32	Physical endurance [title/abstract]
#33	#12 OR #13 OR #14 OR #15 OR #16 OR #17 OR #18 OR #19 OR #20 OR #21 OR #22 OR #23 OR #24 OR #25 OR #26 OR #27 OR #28 OR #29 OR #30 OR #31 OR #32
#34	#11 AND #33

This search strategy will be modified as required for other electronic databases.

**Table 2:** Characteristics of the included studies.

Study	Country	Study type	No. of patients		Interventions		Course of treatment
			PR	Control	PR	Control	
Yun X 2015 [37]	China	RCT	50	50	Exercise training+health education	Routine treatment	12 month
Zhang ZH 2012 [38]	China	RCT	80	80	Exercise training+health education+respiratory training	Routine treatment	2 month
Wang F 2017 [39]	China	RCT	26	25	Exercise training+health education	Routine treatment	2 month
Qu WJ 2017 [40]	China	RCT	41	41	Exercise training+health education	Routine treatment	3 month
Pan W 2017 [41]	China	RCT	30	30	Exercise training+health education+respiratory training	Routine treatment	2 month
Liu QG 2009 [42]	China	RCT	35	25	Exercise training+health education+nutritional guidance+psychological counseling	Routine treatment	3 month
Liu J 2015 [43]	China	RCT	41	41	Exercise training+health education	Routine treatment	3 month
Li XY 2014 [44]	China	RCT	100	100	Exercise training+health education	Routine treatment	2 month
Li F 2015 [45]	China	RCT	40	40	Exercise training+health education+respiratory training+psychological counseling+nutritional guidance	Routine treatment	6 month
Jin YK 2014 [46]	China	RCT	52	50	Exercise training+health education+respiratory training+psychological counseling	Routine treatment	6 month
Cao DF 2010 [47]	China	RCT	30	32	Exercise training+health education+respiratory training	Routine treatment	2 month
Dale 2014 [48]	Australia	RCT	6	4	Exercise training	Routine treatment	4 month
Ma LL 2014 [49]	China	RCT	30	30	Exercise training+respiratory training+psychological counseling+nutritional guidance	Routine treatment	1.5 month
Liu CZ 2014 [50]	China	RCT	30	30	Exercise training+health education+respiratory training	Routine treatment	6 month
Dong QA 2009 [51]	China	RCT	32	32	Exercise training+health education+respiratory training	Routine treatment	3 month
Xiao SY 2019 [52]	China	RCT	32	42	Exercise training+health education+respiratory training	Routine treatment	6 month

**Table 3:** Quality assessment: risk of bias.

Study	Random sequence generation (selection bias)	Allocation concealment (selection bias)	Blinding of participants and personnel (performance bias)	Blinding of outcome assessment (detection bias)	Incomplete outcome data (attrition bias)	Selective reporting (reporting bias)	Other bias
Yun X 2015 [37]	Uncertain risk	Uncertain risk	No	Uncertain risk	Complete	Uncertain risk	Uncertain risk
Zhang ZH 2012 [38]	Uncertain risk	Uncertain risk	No	Uncertain risk	Uncertain risk	Uncertain risk	Uncertain risk
Wang F 2017 [39]	Uncertain risk	Uncertain risk	No	Uncertain risk	Uncertain risk	Uncertain risk	Uncertain risk
Qu WJ 2017 [40]	Uncertain risk	Uncertain risk	No	Uncertain risk	Uncertain risk	Uncertain risk	Uncertain risk
Pan W 2017 [41]	Uncertain risk	Uncertain risk	No	Uncertain risk	Uncertain risk	Uncertain risk	Uncertain risk
Liu QG 2009 [42]	Uncertain risk	Uncertain risk	No	Uncertain risk	Uncertain risk	Uncertain risk	Uncertain risk
Liu J 2015 [43]	Random number table	Uncertain risk	No	Uncertain risk	Uncertain risk	Uncertain risk	Uncertain risk
Li XY 2014 [44]	Uncertain risk	Uncertain risk	No	Uncertain risk	Uncertain risk	Uncertain risk	Uncertain risk
Li F 2015 [45]	Uncertain risk	Uncertain risk	No	Uncertain risk	Uncertain risk	Uncertain risk	Uncertain risk
Jin YK 2014 [46]	Uncertain risk	Uncertain risk	No	Uncertain risk	Uncertain risk	Uncertain risk	Uncertain risk
Cao DF 2010 [47]	Uncertain risk	Uncertain risk	No	Uncertain risk	Uncertain risk	Uncertain risk	Uncertain risk
Dale 2014 [48]	Computer-generated randomization program	Uncertain risk	No	Assessor blind	Complete	No	No
Ma LL 2014 [49]	Uncertain risk	Uncertain risk	No	Uncertain risk	Uncertain risk	Without the primary outcomes	Uncertain risk
Liu CZ 2014 [50]	Lottery	Lottery	No	Uncertain risk	Uncertain risk	Uncertain risk	Uncertain risk
Dong QA 2009 [51]	Uncertain risk	Uncertain risk	No	Uncertain risk	Uncertain risk	Without the primary outcomes	Uncertain risk
Xiao SY 2019 [52]	Random number table	Uncertain risk	No	Uncertain risk	Complete	No	No

**Table 4:** Results of subgroup analysis.

		6MWD (MD, 95% CI)	SGRQ (MD, 95% CI)	FVC (MD, 95% CI)	FVV1 (MD, 95% CI)	FEV1% (MD, 95% CI)	FEV1/FVC (MD, 95% CI)	MVV (MD, 95% CI)
All studies		69.10 [61.95, 76.25]	-9.60 [-16.40, -2.80]	0.20 [0.12, 0.29]	0.23 [0.09, 0.38]	5.19 [1.48, 8.90]	3.61 [-3.43, 10.65]	4.47 [1.14, 7.81]
Intervention subgroup	Exercise training plus health education	64.02 [53.29, 74.76]	-10.16 [-23.40, 3.07]	0.26 [0.03, 0.49]	0.20 [0.12, 0.29]	1.80 [-0.25, 3.85] ∗	-0.61 [-2.30, 1.08]	5.57 [0.38, 10.76]
	Exercise training plus health education plus other measures	73.52 [64.01, 83.04]	-9.47 [-21.77, 2.82]	0.16 [-0.01, 0.33]	0.25 [-0.06, 0.57]	6.03 [2.63, 9.43] ∗	5.30 [-2.72, 13.31]	2.42 [1.34, 3.50]
Time subgroup	≥3 months	67.43 [58.32, 76.54]	-16.84 [-21.45, -12.23]^∗^	0.26 [0.03, 0.49]	0.27 [0.13, 0.41]	5.73 [1.80, 9.66]	/	8.21 [5.07, 11.34]^∗^
	<3 months	71.30 [57.68, 84.92]	-3.64 [-6.20, -1.09]^∗^	0.16 [-0.01, 0.33]	0.15 [-0.28, 0.58]	3.98 [-1.43, 9.39]	/	2.28 [1.25, 3.32]^∗^

^∗^ *P* < 0.05 for subgroup differences.

**Table 5:** Results of sensitivity analysis.

Outcomes	Deletion	Result	
SGRQ	Li XY 2014	*χ* ^2^ = 18.57, *P* < 0.0001, *I*^2^ = 89%	MD-12.05, 95% CI [-21.93, -2.16]
	Yun X 2015	*χ* ^2^ = 8.60, *P* = 0.01, *I*^2^ = 77%	MD-6.61, 95% CI [-12.18, -1.03]
	Li F 2015	*χ* ^2^ = 18.29, *P* = 0.0001, *I*^2^ = 89%	MD-7.80, 95% CI [-15.10, -0.51]
	Zhang ZH 2012	*χ* ^2^ = 20.42, *P* < 0.0001, *I*^2^ = 90%	MD-12.00, 95% CI [-22.09, -1.90]

FVC	Yun X 2015	*χ* ^2^ = 2.15, *P* = 0.14, *I*^2^ = 53%	MD0.16, 95% CI [-0.01, 0.33]
	Ma LL 2014	*χ* ^2^ = 0.10, *P* = 0.75, *I*^2^ = 0%	MD0.22, 95% CI [0.16, 0.29]
	Pan W2017	*χ* ^2^ = 1.78, *P* = 0.18, *I*^2^ = 44%	MD0.15, 95% CI [-0.08, 0.37]

FEV1	Liu J 2015	*χ* ^2^ = 16.88, *P* = 0.0007, *I*^2^ = 82%	MD0.24, 95% CI [0.04, 0.45]
	Qu WJ 2017	*χ* ^2^ = 17.06, *P* = 0.0007, *I*^2^ = 82%	MD0.24, 95% CI [0.04, 0.44]
	Liu CZ 2014	*χ* ^2^ = 10.21, *P* = 0.02, *I*^2^ = 71%	MD0.18, 95% CI [0.04, 0.32]
	Ma LL 2014	*χ* ^2^ = 7.27, *P* = 0.06, *I*^2^ = 59%	MD0.29, 95% CI [0.17, 0.41]
	Pan W2017	*χ* ^2^ = 14.72, *P* = 0.002, *I*^2^ = 80%	MD0.20, 95% CI [0.03, 0.37]

FEV1%	Li XY 2014	*χ* ^2^ = 25.99, *P* < 0.0001, *I*^2^ = 85%	MD6.03, 95% CI [2.63, 9.43]
	Li F 2015	*χ* ^2^ = 6.31, *P* = 0.18, *I*^2^ = 37%	MD3.61, 95% CI [1.66, 5.57]
	Liu CZ 2014	*χ* ^2^ = 66.56, *P* < 0.00001, *I*^2^ = 94%	MD4.94, 95% CI [0.75, 9.14]
	Liu QG 2009	*χ* ^2^ = 60.58, *P* < 0.00001, *I*^2^ = 93%	MD5.72, 95% CI [1.71, 9.74]
	Ma LL 2014	*χ* ^2^ = 67.00, *P* < 0.00001, *I*^2^ = 94%	MD4.83, 95% CI [0.71, 8.95]
	Xiao SY 2019	*χ* ^2^ = 55.13, *P* < 0.00001, *I*^2^ = 93%	MD5.49, 95% CI [1.16, 9.82]

FEV1/FVC	Qu WJ 2017	*χ* ^2^ = 437.78, *P* < 0.00001, *I*^2^ = 99%	MD4.24, 95% CI [-3.37, 11.84]
	Yun X 2015	*χ* ^2^ = 399.21, *P* < 0.00001, *I*^2^ = 99%	MD4.39, 95% CI [-3.05, 11.82]
	Jin YK 2014	*χ* ^2^ = 472.57, *P* < 0.00001, *I*^2^ = 99%	MD3.63, 95% CI [-4.47, 11.72]
	Li F 2015	*χ* ^2^ = 14.83, *P* = 0.005, *I*^2^ = 73%	MD1.67, 95% CI [-0.79, 4.13]
	Liu CZ 2014	*χ* ^2^ = 398.03, *P* < 0.00001, *I*^2^ = 99%	MD3.50, 95% CI [-4.76, 11.76]
	Liu J 2015	*χ* ^2^ = 301.39, *P* < 0.00001, *I*^2^ = 99%	MD5.29, 95% CI [-2.72, 13.31]
	Xiao SY 2019	*χ* ^2^ = 307.20, *P* < 0.00001, *I*^2^ = 99%	MD5.37, 95% CI [-2.60, 13.34]

MVV	Li XY 2014	*χ* ^2^ = 11.70, *P* = 0.003, *I*^2^ = 79%	MD5.87, 95% CI [1.26, 10.49]
	Liu J 2015	*χ* ^2^ = 7.30, *P* = 0.03, *I*^2^ = 73%	MD3.38, 95% CI [0.07, 6.69]
	Qu WJ 2017	*χ* ^2^ = 7.27, *P* = 0.03, *I*^2^ = 72%	MD3.38, 95% CI [0.07, 6.68]
	Zhang ZH 2012	*χ* ^2^ = 9.32, *P* = 0.009, *I*^2^ = 79%	MD5.57, 95% CI [0.38, 10.76]

**Table 6:** GRADE summary of primary outcomes.

PR compared to routine treatment for pneumoconiosis						
Patient or population: patients with pneumoconiosis						
Settings:						
Intervention: PR						
Comparison: routine treatment						
Outcomes	Illustrative comparative risks^∗^ (95% CI)		Relative effect (95% CI)	No. of participants (studies)	Quality of the evidence (GRADE)	Comments
	Assumed risk	Corresponding risk				
	Routine treatment	PR				
6MWD	The mean 6MWD ranged across control groups from -0.88 to 58.26	The mean 6MWD in the intervention groups was 69.10 higher (61.75 to 76.25 higher)		1049 (12 studies)	⊕⊕⊕⊝ moderate^1^	
SGRQ	The mean SGRQ ranged across control groups from -1.79 to -0.02	The mean SGRQ in the intervention groups was 9.6 lower (16.4 to 2.8 lower)		540 (4 studies)	⊕⊝⊝⊝ very low^1,2^	

^∗^The basis for the assumed risk (e.g., the median control group risk across studies) is provided in footnotes. The corresponding risk (and its 95% confidence interval) is based on the assumed risk in the comparison group and the relative effect of the intervention (and its 95% CI). CI: *c*onfidence interval; GRADE Working Group grades of evidence. High quality: further research is very unlikely to change our confidence in the estimate of effect. Moderate quality: further research is likely to have an important impact on our confidence in the estimate of effect and may change the estimate. Low quality: further research is very likely to have an important impact on our confidence in the estimate of effect and is likely to change the estimate. Very low quality: We are very uncertain about the estimate. ^1^No blind method and assignment concealment. ^2^High heterogeneity (*I*^2^ = 88%) was found.

## Supplementary Materials

Supplementary 1 Table S1: PRISMA checklist.

Supplementary 2 Figure S1: subgroup analysis of intervention for 6MWD.

Supplementary 3 Figure S2: subgroup analysis of intervention for SGRQ.

Supplementary 4 Figure S3: subgroup analysis of intervention for FVC.

Supplementary 5 Figure S4: subgroup analysis of intervention for FEV1.

Supplementary 6 Figure S5: subgroup analysis of intervention for FEV1%.

Supplementary 7 Figure S6: subgroup analysis of intervention for FEV1/FVC.

Supplementary 8 Figure S7: subgroup analysis of intervention for MVV.

Supplementary 9 Figure S8: subgroup analysis of duration for 6MWD.

Supplementary 10 Figure S9: subgroup analysis of duration for SGRQ.

Supplementary 11 Figure S10: Subgroup analysis of duration for FVC.

Supplementary 12 Figure S11: subgroup analysis of duration for FEV1.

Supplementary 13 Figure S12: subgroup analysis of duration for FEV1%.

Supplementary 14 Figure S13: subgroup analysis of duration for MVV.
